# Analysis of TGA data for polyvinyl alcohol at slow heating rate using deep neural networks, activation energy, and activation enthalpy

**DOI:** 10.1038/s41598-025-22946-8

**Published:** 2025-10-29

**Authors:** Abdulrazak Jinadu Otaru, Zaid Abdulhamid Alhulaybi Albin Zaid

**Affiliations:** Department of Chemical Engineering, College of Engineering, King Faisal University, Al Ahsa, 31982 Saudi Arabia

**Keywords:** Polyvinyl alcohol, TGA data, Deep neural networks, Activation energy, Activation enthalpy, Chemical engineering, Environmental impact

## Abstract

This research aims to enhance the deep neural network (DNN) model previously developed by this group, as referenced in^10^, by integrating degradation time into its feature set. It specifically addresses the thermal decomposition of polyvinyl alcohol (PVA) at low heating rates of 2, 5, and 10 °C.min^-1^. In addition, the study presents a thermo-kinetic analysis of the data, facilitating the estimation of activation energy and activation enthalpy. The inputs to the DNN frameworks include degradation time, degradation temperature, and heating rate. Modifications were made to the DNN model to tackle overfitting and reduce the discrepancy between output signals and experimental scatter. This was accomplished through iterative adjustments to the learning rate, implementation of data augmentation techniques, prolongation of the training duration, and early termination to minimize error. An optimized DNN architecture, comprising two hidden layers and eight neurons, effectively facilitated the learning algorithms and successfully trained arbitrary constants. This resulted in output signals that closely aligned with the experimental data ($\:{R}^{2}\sim0.999$), thereby providing a ranking of parameter sensitivity characterized by heating rate, time, and degradation temperature. The Flynn-Wall-Ozawa (FWO) and Kissinger-Akahira-Sunose (KAS) model-free equations were used to estimate the activation energy ($\:{E}_{A}$) of the thermogravimetric (TGA) data curves. The estimated average $\:{E}_{A}$ values derived from the FWO and KAS model-free equations were 64.6±3.2 kJ·mol⁻¹ and 58.8±2.9 kJ·mol⁻¹, respectively, based on conversion rates between 5 and 50 wt% (i.e., where $\:{R}^{2}>0.9$). The estimated theoretical value of the activation enthalpy (ΔH) required for the formation of the activation complex, at these higher correlations, was determined to be positive (25.4– 102.0 kJ·mol⁻¹), indicating that the reaction is invariably endothermic and is consistent with established information in the literature. This approach could prove pivotal for manufacturers in the design and fabrication of polyvinyl alcohol (PVA) and composites with enhanced and novel properties.

## Introduction

Polymeric materials play a significant role in various aspects of our daily lives, including homes, schools, auditoriums, and industries. These materials are renowned for their lightweight nature and are primarily manufactured by petrochemical industries worldwide through the polymerization process, wherein monomers or repeating units are linked together within the molecular structure^1^. The distinct combination of lightweight composition, high stiffness, and inherent ability to form large molecules allows for their extensive use in diverse applications such as food packaging, medical and laboratory equipment, computer hardware, automobiles, and aerospace, among others. However, despite the numerous advantages offered by these materials, they possess non-biodegradable properties and can only degrade through direct pyrolysis or with the introduction of biodegradable catalysts under specific heat conditions. Therefore, a comprehensive understanding of the thermal stability and degradation behaviour of these materials is crucial for designing polymers and composites with enhanced properties, ensuring optimal applications, and minimizing energy requirements for their decomposition.

The thermal decomposition of polymeric materials has been extensively studied, focusing on various operating conditions and material variations. Most studies have relied on experimental approaches. For instance, Alhulaybi and Dubdub^2^ examined the kinetics and thermodynamics of polyvinyl alcohol (PVA) using heating rates of 20, 30, and 40 °C.min⁻¹, applying both model-free and model-fitting kinetic models. They determined an average activation energy of 126 kJ.mol⁻¹ for the conversion of 10% to 70% of the TGA traces at different heating rates. Another study investigated the thermal decomposition kinetics of polyethylene terephthalate (PET)^3^ under inert conditions, revealing that the degradation rate ($\:{R}_{D}$), activation energy ($\:{E}_{A}$), and pre-exponential factor ($\:A$) depended significantly on the heating rate, while the reaction order was found to be constant and equal to one across all TGA data curves.

Chowdhurry and Wang^4^ investigated the thermal decomposition of PET using the Friedman model-free kinetics. They examined different heating rates of 10, 20, and 30 °C.min^−1^ and employed model-fitting equations (Arrhenius and Coats-Redfern) designed for constant heating rates. The thermodynamics of the degradation process were reported as non-spontaneous and endothermic for all TGA traces. The study in^4^ also applied machine learning tools to predict the weight loss profile resulting from the pyrolysis of PET microplastics, which will be discussed in subsequent sections. Wulandari et al.^5^ conducted an experimental study on the thermal decomposition of PVA and PVA/cassava starch (CS)/alkaline lignin (AL) using a TGA system with a heating rate of 10 °C.min^−1^. They determined the activation energy ($\:{E}_{A}$) values of 57.1, 64.8, and 70.6 kJ.mol^−1^ using the Coats-Redfern, Briodo, and Horowitz and Merger kinetic models, respectively. The $\:{E}_{A}$ value for PVA/CS/AL was found to be higher than that for pure PVA, indicating that the composites exhibited higher thermal stability. Another related study^6^ reported that the thermal stability of starch/PVA was lower than that of pure starch, and the thermal stability of the composites decreased with increasing PVA content. The estimated activation energy values for the composites were 76 kJ·mol⁻¹ and 81 kJ·mol⁻¹ when applying the Kissinger-Akahira-Sunose (KAS) and Flynn-Wall-Ozawa (FWO) model-free kinetic models, respectively, indicating a strong concordance between the two models.

Recent advancements in research and innovation in the thermal decomposition of polymers and composites have also involved the use of machine learning modelling and simulation techniques of artificial intelligence (AI). These techniques have been used to develop learning algorithms that can predict the TGA traces of these materials under similar operating conditions as the experimental conditions. For example, Conesa et al.^7^ demonstrated that a well-trained multilayered neural network can reliably predict the experimental TGA data curves of different polymer materials at various heating rates. In a study conducted by^3^, it was found that a two-layer hidden architecture of an artificial neural network could accurately predict ($\:{R}^{2}\to\:0.95-1.00$) the TGA data for PET microplastics. The study in^4^ also utilized traditional deep neural networks (DNN) for predicting the weight loss profile resulting from the degradation of this material. It was found that a two-layer hidden architecture yielded the most appropriate model, with a regression coefficient value ranging from 0.95 to 1.00. Alhulaybi and Otaru^8^ utilized a modified 2-hidden layer deep neural network (DNN) architecture to develop learning algorithms that accurately forecasted the thermal decomposition of Phoenix Dactylifera/HDPE composites. The predictions successfully overlapped with experimental results for a range of degradation temperatures (25 to 600 °C) and heating rates (10, 20, and 40 °C.min^−1^), resulting in a high correlation coefficient. Similarly, a comparable 2-hidden layer DNN framework was employed in a study by^9^ to predict the thermal stability and degradation of HZSM-5/HDPE composites at heating rates of 5, 10, and 15 °C.min^−1^.

A recent study (reference^10^ conducted an experimental and machine learning DNN analysis on polyvinyl alcohol (PVA) thermogravimetric traces at low heating rates of 2, 5, and 10 °C.min⁻¹. This study highlighted the influence of heating rate and degradation temperature (input signals) on the weight (output) of PVA. However, it did not address the effect of degradation time or the kinetics of the material’s decomposition mechanisms. Degradation time is a critical parameter that could improve the understanding of potential reaction pathways and contribute to the hierarchical ordering of process parameters on the pyrolysis of this material. To address this gap, the present work utilized machine learning DNN techniques to develop algorithms for predicting the thermal decomposition of PVA at low heating rates of 2, 5, and 10 °C.min⁻¹, incorporating degradation time as part of the input parameters. Additionally, this work explored model-free and model-fitting kinetic models to estimate the activation energy and enthalpy of both the experimental and predicted TGA data curves under various heating conditions.

It is essential to note that Saudi Arabia hosts several petrochemical industries producing plastic materials for both domestic use and export, significantly contributing to the nation’s gross domestic product. The ongoing production of these polymeric materials has led to increased plastic waste in landfills, prompting the establishment of the Saudi Green Initiative to develop more efficient waste management strategies. Furthermore, many studies on the degradation of polyvinyl alcohol (PVA) materials have focused primarily on high heating rates, with limited attention to degradation at lower heating rates. Cetin et al.^11^ characterized high heating rate degradation as fast pyrolysis, often resulting in rapid phase transformation and significant material loss over a short time. In contrast, Albin Zaid and Otaru^12^ observed that slow pyrolysis typically leads to longer degradation times, due to the predominant influence of pyrolysis temperature on the materials. Consequently, incorporating degradation time into the modelling and simulation of the weight loss profile during polymer degradation is crucial, as is estimating the underlying reaction mechanisms, kinetics, and thermodynamics involved.

## Machine learning DNN and data

**Fig. 1:**
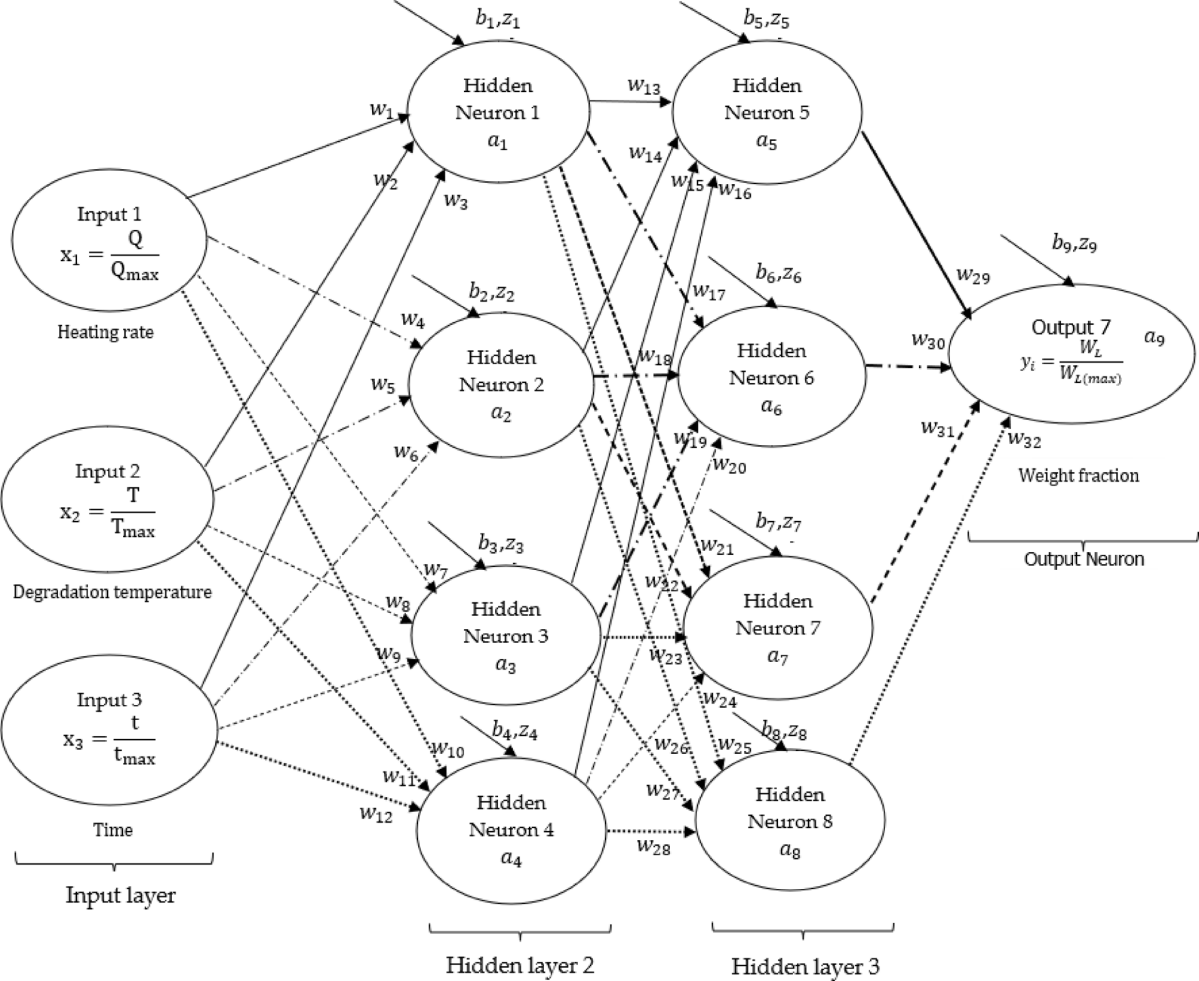
Illustration of DNN framework showing typical input, hidden and output layers consisting of neurons (that is, eight hidden neurons or simply 8HNS).

The term “deep neural networks” refers to a specific category of traditional artificial neural networks (ANNs) or deep learning methodologies, characterized by the presence of input, output, and multiple hidden neurons and layers. This complexity renders deep neural networks more resource-intensive compared to convolutional neural networks^13^. The backpropagation method employed in machine learning deep neural network (DNN) was utilized in this study to develop learning algorithms aimed at understanding the correlation between weight loss (output/response) of the PVA and various input parameters, specifically, heating rate ($\:{Q}_{R}$), degradation temperature (T), and degradation time (t). Figure 1 illustrates a DNN framework that incorporates these three input parameters, two hidden layers, and an output response, all connected through 32 synaptic weights ($\:{w}_{i}$) and 9 biases ($\:{b}_{i}$). A similar technique, as described in^9,10,14–16^, was employed to design the DNN architecture and its learning algorithms utilizing the backpropagation method. Furthermore, the rationale for selecting this framework (Fig. 1) is informed by findings in^4,7^, which suggest that a two-hidden-layer architecture is an optimal choice for the modelling and simulation of weight loss during the thermal degradation of polyethylene terephthalate (PET)^4^ and various selected polymeric materials^7^. The initial selection of eight hidden neurons during the training process was predicated on the need to avoid an excessive number of hidden neurons, which could potentially lead to prolonged training times and poor future data predictions due to overfitting^15,17^. Conversely, selecting too few hidden neurons may result in inadequate predictions of the experimental data, leading to underfitting^15,18^.

The technique employed in this study for developing learning algorithms for the Deep Neural Network (DNN) framework (as shown in Fig. 1) begins with the output response and proceeds through the 2 hidden layers to the input signals. This approach, known as backpropagation^14,16^, involves utilizing the general Artificial Neural Networks (ANN) model described in Eq. 1. To move from the output to the input, it is necessary to determine the activation function for each neuron within the DNN framework. Although various mathematical models have been proposed in previous work^15^ for estimating this activation function, the widely used Sigma activation function (Eq. 2) is selected in this study to formulate the learning algorithm due to its predictive accuracy in accurately representing values between 0 and 1^15,19–21^. Therefore, the input and output signals utilized for training purposes were normalized by dividing them by their corresponding maximum experimental values. Specifically, values of 10 °C.min^−1^, 600 °C, and 290 min were selected to represent the highest achievable experimental data for heating rate, degradation temperature, and degradation time, respectively. Furthermore, a weight loss value of 100% was chosen as the maximum attainable value, typically observed at the initiation of the TGA process before any loss of moisture or material constituents occurs.

Before optimizing the framework illustrated in Fig. 1 to enhance its predictive accuracy, it is imperative to first assess the response of the formulated learning algorithms in capturing the behaviour characterized by the PVA thermogravimetric traces at the selected heating rates. This assessment is essential for the optimal utilization of computational resources, time, and modelling accuracy, while also drawing upon existing literature and machine learning modelling expertise. It is important to recognize that the efficacy of a predictive model is contingent upon its ability to accurately generate responses that correlate with experimental outcomes. However, achieving 100% accuracy in modelling presents significant challenges; thus, it is necessary to consider residual errors ($\:{R}_{E}$) or the cost function (*C*), which quantifies the disparity between the actual values ($\:y$) and the predictions ($\:a$) as defined by Eq. 3. Enhancing the predictive accuracy of learning algorithms within the DNN framework (Fig. 1) was achieved through the development of cost (loss) optimization algorithms that aim to minimize the discrepancy between predictions and experimental results. These algorithms were formulated by analyzing the dependency of the cost function on the output, hidden, and input neurons in a backpropagation sequence. Such algorithms are crucial for modifying the weights of the connecting neurons within the DNN framework to minimize prediction error^19^. Alternatively, reducing the cost function in Eq. 1 could involve a trial-and-error approach of predicting the arbitrary constants (synaptic weights and biases) as mentioned in^14^. However, this approach is labour-intensive, exhausting, and may yield minimal to no significant improvement in modelling accuracy^14,16^.

The significance of both the quality and quantity of data needed for the training of deep neural network (DNN) learning algorithms is paramount. Research conducted in^9,20^ indicates that a substantial volume of experimental data can enhance the accuracy of the trained model and facilitate convergence. In this study, three datasets—namely heating rate, degradation temperature, and degradation time—were utilized as input variables, alongside a singular output dataset representing weight loss, to train the learning algorithms within the DNN framework (see Fig. 1). These datasets comprised approximately 177 data points, which were systematically selected from the 27,603 raw experimental data points obtained through thermogravimetric analysis (TGA) experimentation. The remaining data points were allocated for validation purposes. After training the selected experimental datasets utilizing the DNN framework illustrated in Fig. 1, which comprises eight hidden neurons, analogous data were subsequently trained using a modified iteration of the DNN framework presented in the same figure. The modification involved reducing the number of hyperparameters, specifically the hidden neurons, to achieve optimal arbitrary constants that would minimize residual error and computational demand. This was accomplished by removing one hidden neuron ($\:{a}_{8}$) from the original framework, resulting in a model with seven hidden neurons (i.e., the DNN [7 HNS]). Subsequently, learning algorithms for this framework were formulated and computed. Additionally, two hidden neurons ($\:{a}_{4}\:\&\:{a}_{8}$) were also removed from the original DNN framework depicted in Fig. 1, leading to a two-hidden layer architecture with six hidden neurons (i.e., DNN [6 HNS]). Learning algorithms and computations were then developed for this modified architecture.

The selection and optimization of hidden layers and neurons within the deep neural network (DNN) architecture are essential for effectively capturing the complex relationships between the input parameters—namely degradation temperature, degradation time, and heating rates—and the output label, which consists of thermogravimetric analysis (TGA) traces or weight loss. Furthermore, it is imperative to mitigate the risks of both overfitting and underfitting. Notably, the decision to employ a configuration of two hidden layers, each containing eight neurons, as part of the initial hyperparameters for DNN modelling and simulation in this study is informed by the findings presented in references^4,9^. Al-Yaari and Dubdub^9^ concluded that a DNN framework comprising two hidden layers with ten neurons each serves as a suitable foundation for training thermogravimetric (TGA) traces, configured within a LogSig-TanSig framework. Additionally, Chowdhurry and Wang^4^ suggests that a two-hidden layer architecture is optimal for predicting TGA curves associated with the thermal devolatilization of polyethylene terephthalate (PETE). While the initial selection of two hidden layers with eight neurons is noteworthy, it is crucial to highlight that the determination of the optimal architecture for this study involved a trial-and-error methodology, wherein the hyperparameter value was iteratively adjusted until the predicted model closely aligned with the experimental measurements.

1 $$\:Z=\sum\:_{i}{w}_{i}.{x}_{i}+{b}_{k}$$

2 $$\:a=\sigma\:{\prime\:}\left[z\right]=\:\frac{1}{1+{e}^{-z}}$$

 

3 $$\:C={(y-a)}^{2}$$

 

**Fig. 2:**
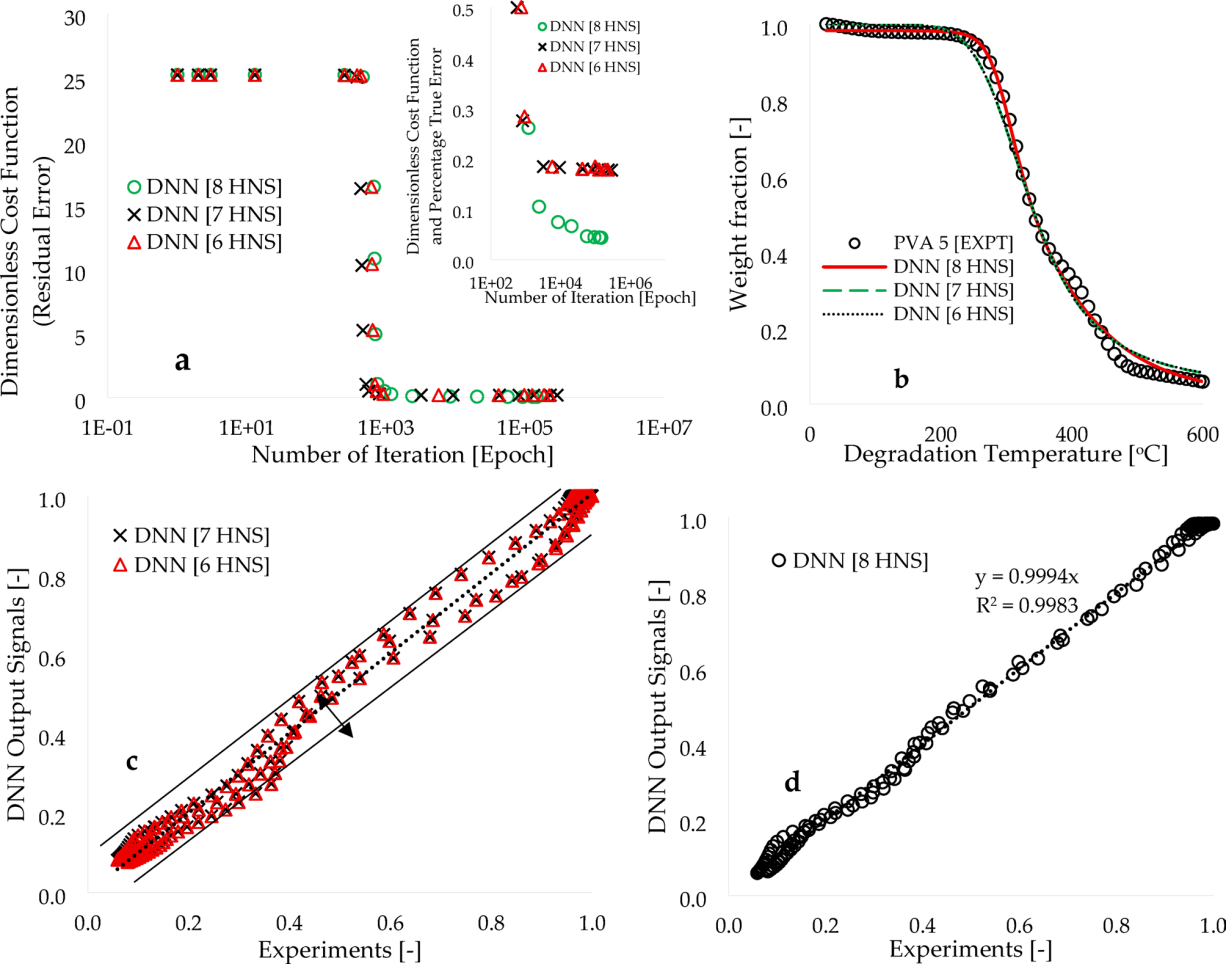
Plots of (**a**) Residual error [-] against epoch [-], (**b**) experimental and DNN computed weight fraction [-] against degradation temperature [^o^C], (**c**) DNN output signals [-] against experiment for the 6 and 7 HNS and (**d**) DNN output signals [-] against experiment [-].

Figure 2a illustrates plots of the overall cost function (C) or residual error ($\:{R}_{E}$) as a function of the number of computation iterations (also known as epochs) employed for the trained model. Initially, a value of 0.1 was consistently assigned to all synaptic weights and biases, resulting in cost functions of 25.238, 25.311, and 25.316 for the DNN [8 HNS], DNN [7 HNS], and DNN [6 HNS] frameworks, respectively. Subsequent training of learning algorithms for these frameworks was conducted using a Visual Basic for Application (VBA) code, with an initial time step of one second employed for the complete computational process. The computational time was further minimized to zero to expedite the process and achieve convergence within shorter time intervals, while still targeting accuracy. Figure 2a illustrates a significant decrease in the initial cost function response achieved by training the learning algorithms developed for the DNN [8 HNS]. The value reached was 0.043, corresponding to a true error ($\epsilon_{\text{T}}$) of 0.172%. This reduction was accomplished through a time investment of 4.78 h and approximately 146,777 epochs. Furthermore, when the learning algorithms designed for DNN [7 HNS] and DNN [6 HNS] were used to train the experimental data, estimated true error values of 0.70% and 0.74% were obtained, respectively. It is worth noting that these values were obtained through a longer computational period and twice the number of epochs compared to the DNN [8 HNS] framework.

Figure 2b presents the comparison between the DNN output data and the TGA experimental data carried out with a heating rate of 5 °C.min^−1^. The figure reveals a complete overlap between the modelling and experimental data, highlighting the validity of well-trained machine learning algorithms in accurately describing the behaviour and response of a process based on input data. In Fig. 2c, the deviation between the modelling (DNN [7 HNS] and DNN [6 HNS]) and experimental results is shown for 177 data points. The coefficient of determination ($\:{R}^{2}$) is found to be 0.9931 and 0.9932 for the DNN [7 HNS] and DNN [6 HNS], respectively. Conversely, Fig. 2d demonstrates a stronger alignment between the modelling using learning algorithms developed for the DNN [8 HNS] framework and the experiment, resulting in a higher coefficient of determination ($\:{R}^{2}$) of 0.9995. Furthermore, Table 1 provides statistical comparisons between the modelled and experimental data, demonstrating significantly improved correlations in terms of mean value, percentage estimate uncertainty (Δ), and mean absolute error (MAE) for the DNN [8 HNS] in relation to the experimental data. The DNN [6 HNS] achieves a residual error of 0.74%, requiring 6.4 h of computational time and 235,515 epochs. On the other hand, the DNN [7 HNS] achieves a slightly lower residual error of 0.70%, but at the cost of approximately 8.06 h and 289,414 epochs.

**Table 1:** Tabular representation of trained data estimated for the three different DNN frameworks.

	b_1_	w_1_	w_2_	w_3_	b_2_	w_4_	w_5_	w_6_	b_3_		w_7_		w_8_	
DNN [8 HNS]	2.4014	0.2076	−3.2579	0.2459	2.4014	0.2076	−3.2579	0.2459	2.4014		0.2076		−3.2579	
DNN [7 HNS]	−0.7912	0.2525	−2.785	−0.0426	−0.7912	0.2525	−2.785	−0.0426	−0.7912		0.2525		−2.785	
DNN [6 HNS]	−0.412	0.2751	−3.0418	−0.0474	−0.412	0.2751	−3.0418	−0.0474	−0.412		0.2751		−3.0418	

	w_9_	b_4_	w_10_	w_11_	w_12_	b_5_	w_13_	w_14_	w_15_		w_16_		b_6_	
DNN [8 HNS]	0.2459	7.8334	0.1394	−16.827	−7.2685	−2.687	1.3195	1.3195	1.3195		4.198		−2.687	
DNN [7 HNS]	−0.0426	−0.7912	0.2525	−2.785	−0.0426	−2.4869	3.0171	3.0171	3.0171		3.0171		−2.4869	
DNN [6 HNS]	−0.0474	0	0	0	0	−2.273	3.1898	3.1898	3.1898		0		−2.273	

	w_17_	w_18_	w_19_	w_20_	b_7_	w_21_	w_22_	w_23_	w_24_	b_8_	w_25_	w_26_	w_27_	w_28_
DNN [8 HNS]	1.3195	1.3195	1.3195	4.198	−2.687	1.3195	1.3195	1.3195	4.198	−2.687	1.3195	1.3195	1.3195	4.198
DNN [7 HNS]	3.0171	3.0171	3.0171	3.0171	−2.4869	3.0171	3.0171	3.0171	3.0171	0	0	0	0	0
DNN [6 HNS]	3.1898	3.1898	3.1898	0	−2.273	3.1898	3.1898	3.1898	0	0	0	0	0	0

	b_9_	w_29_	w_30_	w_31_	w_32_	R_E_	I_N_	ε_A_	ε_T_	L	R^2^	Mean	[%]	MAE
DNN [8 HNS]	−4.5765	2.1725	2.1725	2.1725	2.1725	0.0433	146,777	1.203	0.1717	5	0.9995	0.5901	2.8433	0.3565
DNN [7 HNS]	−5.0464	8.194	8.194	8.194	0	0.1772	289,404	0.1063	0.7003	2	0.9932	0.5945	2.8639	0.3589
DNN [6 HNS]	−5.0451	7.0303	7.0303	7.0303	0	0.1782	223,515	0.0222	0.7037	0.5	0.9931	0.5947	2.862	0.3591
											EXPT	0.5903	2.845	0.357

$\:{\text{w}}_{\text{i}}\:\text{i}\text{s}\:\text{w}\text{e}\text{i}\text{g}\text{h}\text{t},{\text{b}}_{\text{i}}\:$is bias,$\:{\:\text{R}}_{\text{E}}\:$is residual error, $\:{\text{I}}_{\text{N}}$ number of iterations, $\epsilon_{\text{A}}$ is approximate error, $\epsilon_{\text{T}}$ true error and$\:\text{L}$ is learning rate.

Table 1 displays the calculated values of biases ($\:{b}_{i}$), synaptic weights ($\:{w}_{i}$), residual error ($\:{R}_{E}$), approximate error ($\epsilon_{\text{A}}$), true error ($\epsilon_{\text{T}}$), and the learning rate ($\:L$) utilized in the training of the learning algorithms developed for the three DNN frameworks. An unchanging learning rate of 5.0 was maintained consistently throughout the computation involving the DNN [8 HNS] framework, while the learning rates of the DNN [7 HNS] and DNN [6 HNS] were adjusted from 5.0 to 2.0 and from 5.0 to 0.5, respectively. This adjustment in the learning rate is crucial for modifying the modelling response during the training process. At a certain stage in the training process, the difference between the consecutive residual errors or cost functions becomes negligible and, in some cases, starts to increase - which contradicts the objective of the cost optimization function. At this point, the training was prematurely stopped by reducing the learning rate and significantly enhancing the overall modelling responses for the two frameworks.

It is essential to emphasize that the implementation of modelling regularization through the systematic reduction of learning rates and extended training durations enabled the methodical adjustment of derivative functions, synaptic weights, and biases computed in the direction of negative gradients, while ensuring that these gradients were not trapped in local minima^22^. This approach contributed to a decrease in training time and the number of epochs, while simultaneously enhancing the convergence of the model toward a complete alignment between modelling data and experimental measurements. While biases provide an offset in the overall output signals during the training process, the estimated value of a synaptic weight indicates the contributing role of an input signal to an activation function^8,16^. A higher positive value of the synaptic weight indicates a greater contribution of the resulting input response to the output activation, whereas a negative synaptic weight value signifies weak input data for the overall response. Table 1 demonstrates that, with the optimal DNN [8 HNS] framework, the positive values of $\:{w}_{1}$ and $\:{w}_{3}$ indicate that heating rate and degradation time (a variable newly introduced in this study in comparison to the previous report in^10^ have a more influential role in accurately describing the resulting output weight loss of the PVA material during degradation.

**Fig. 3:**
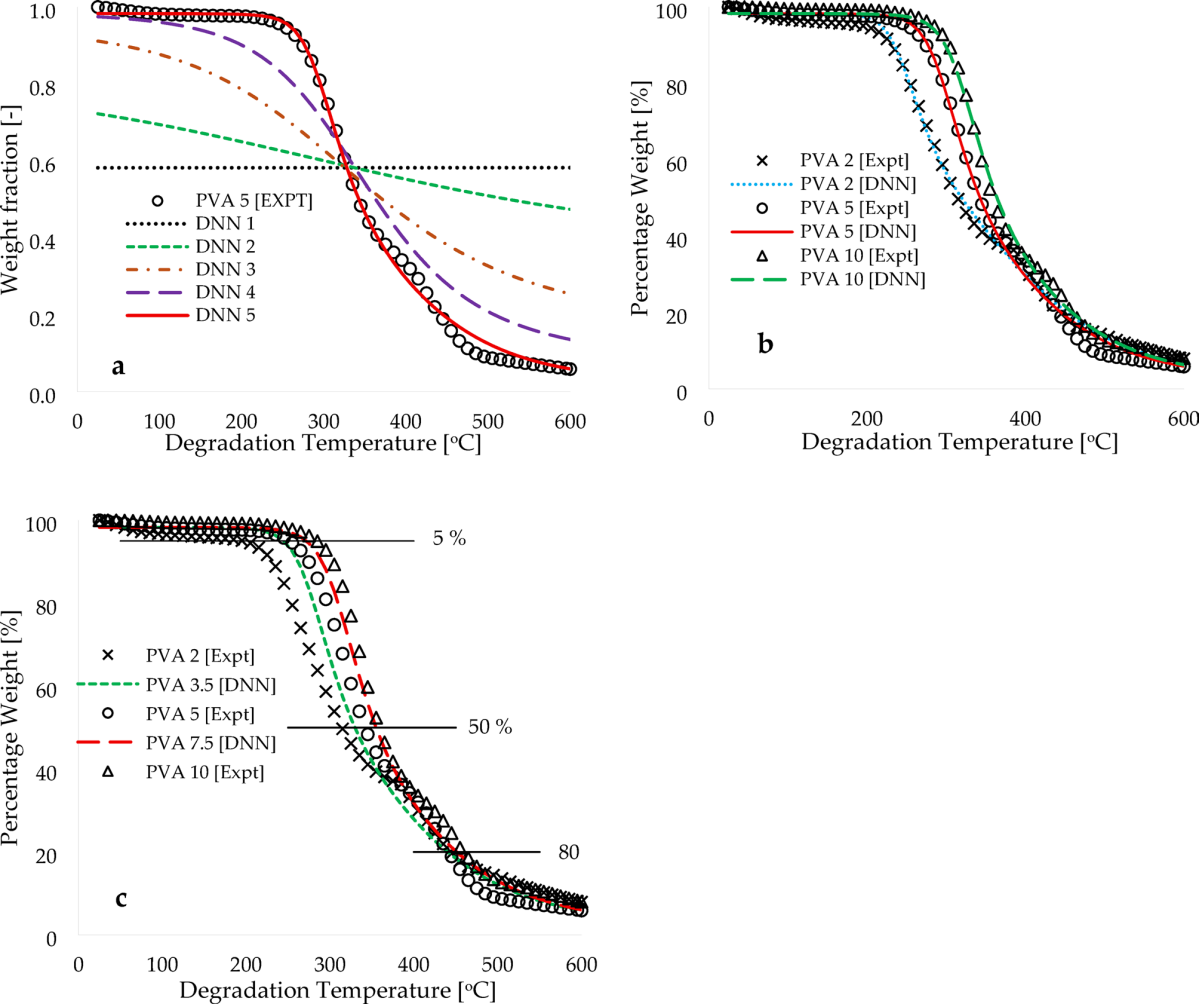
Plots of experimental and DNN modelled (**a**) weight fraction [-], (**b**) percentage weight and (**c**) predicted percentage weight against degradation temperature.

Figure 3 depicts the comparison between the DNN model and the selected experimental TGA data. The TGA data was obtained at a heating rate of 5 °C.min^−1^, with degradation temperatures ranging from 25 to 600 °C. This comparison was done to validate the model. In Fig. 3a, five predicted signals are plotted, representing different stages of the training process. These plots show a gradual improvement in the predicted values, starting from an initial fixed value of 0.1 and reaching the final computed constants. The cost function also shows a gradual decline from 25.238 (DNN1) to 0.172 (DNN5). Observably, the final predicted values (DNN5) exhibit a complete overlap with the experimental data for the entire range of degradation temperatures, displaying a coefficient of correlation that is close to unity. Figure 3b illustrates the plots of the DNN modelled data and the experimental data for the PVA materials at heating rates of 2, 5, and 10 °C.min^−1^. These plots demonstrate a complete overlap, providing evidence of the accurate distribution of the modelling across the scatter of experimental results, with a correlation exceeding 99%. In addition, the strength of the learning algorithms and trained arbitrary constants was tested using the actual experimental operating heating rates.

Figure 3c shows the DNN predicted values of TGA data curves at 3.5 and 7.5 °C.min^−1^ plotted against degradation temperature. These plots follow the typical trends of TGA traces found in the literature^3–8,23–26^ and in this study - that is, a shift in the TGA data curve towards higher temperatures with increasing heating rate. Table 2 displays the thermal data obtained from both the experimental and predicted TGA data curves, revealing a consistent increase in temperature with elevated heating rates. For example, at a weight% of 50%, the temperature values recorded are 314, 330, 342, 355, and 359 °C for the TGA data curves corresponding to heating rates of 2.0, 3.5, 5.0, 7.5, and 10.0 °C.min⁻¹, respectively. It is important to highlight that the predicted TGA data curve at 3.5 °C·min⁻¹ is situated between the experimentally measured TGA traces at heating rates of 2 and 5 °C·min⁻¹. Similarly, the predicted TGA data curve at 7.5 °C·min⁻¹ is positioned between the experimentally measured TGA traces at 5 and 10 °C·min⁻¹. This observation underscores the sensitivity of the learning algorithms, as well as the computed arbitrary constants, in accurately characterizing the thermal degradation of polyvinyl alcohol (PVA) materials at lower heating rates, which typically range from 2 to 10 °C·min⁻¹. The modelling output, along with experimental data, can be used to estimate kinetics and thermodynamics parameters, which in turn can help understand the reaction pathway and heat requirements for the pyrolysis of PVA at these slow heating rates.

**Table 2:** Thermal data from the TGA data curves.

Thermograms	Initial	50 wt%	Final	T_max_
PVA 2 [Expt]	25 °C	314 °C	600 °C	445 °C
	7.81 mg	3.91 mg	0.62 mg	
PVA 3.5 [DNN]	25 °C	330 °C	600 °C	435 °C
PVA 5 [Expt]	25 °C	342 °C	600 °C	440 °C
	7.69 mg	3.85 mg	0.45 mg	
PVA 7.5 [DNN]	25 °C	355 °C	600 °C	450 °C
PVA 10 [DNN]	25 °C	359 °C	600 °C	455 °C
	7.10 mg	3.55 mg	0.57 mg	

NB: $\:{T}_{max}$ is the maximum temperature of constant conversion (80 wt%).

## Activation energy and activation enthalpy

As part of the study on kinetics and thermodynamics of the process, the activation energy and activation enthalpy were estimated both at a constant heating rate and constant conversions. To estimate the activation energy, the Flynn-Wall-Ozawa (FWO) model-free method (Eq. 1) and the Kissinger-Akahira-Sunose (KAS) model-free method for variable heating rate (Eq. 2) were employed^6,27,28^. Equation 3 presents a mathematical relationship between the activation enthalpy ($\:\varDelta\:H\:$in kJ.mol^−1^) as a test function of the activation energy ($\:{E}_{A}$ in kJ.mol^−1^)^29,30^, gas constant (*R* in kJ.mol^−1^.K^−1^), and the maximum temperature of constant conversion ($\:{T}_{Max}$ in K). It is crucial to note that the activation enthalpy ($\:\varDelta\:H$) is associated with the formation of activation complex (i.e. transition or intermediate state) from the reactant. This is regarded^30^ as an enthalpic barrier to the reaction, which is consistently positive (indicating an endothermic reaction). This stands in contrast to the enthalpy of conversion of the reactant to the product ($\:{\varDelta\:H}_{rxn})$^30^. In summary, Eq. 3 does not offer any insights into the free energy change associated with the conversion of reactants to products.

**FWO model-free method for variable heating rate**

 

4 $$\:\text{l}\text{n}\left({Q}_{R}\right)=\text{ln}\left(\frac{A.{E}_{A}}{R.\left(-\text{l}\text{n}(1-{x}_{i}\right)}\right)-5.331-1.052\left(\frac{{E}_{A}}{R}\right).\frac{1}{{T}_{c}}$$

**KAS model-free method for variable heating rate**

 

5 $$\:\text{ln}\left({Q}_{R}/{T}_{C}^{2}\right)=\text{ln}\left(\frac{A.R}{{E}_{A}.\left(-\text{l}\text{n}(1-{x}_{i}\right)}\right)-\left(\frac{{E}_{A}}{R}\right).\frac{1}{{T}_{c}}$$

## Activation enthalpy

 

6 $$\:\varDelta\:H={E}_{A}-R.{T}_{Max}$$

where $\:{w}_{o}$ is initial sample weight, $\:{w}_{t}$ is weight at time t, $\:{w}_{f}$ is the final sample weight, $\:{T}_{c}$ is the temperature of constant conversion [K], $\:{Q}_{R}$ is the heating rate [^o^C.min^−1^], $\:A$ is pre-exponential factor and $\:t$ is the degradation time (sec). The application of the FWO and KAS isoconvensional model-free methods for multiple heating rates in this study, is intended to facilitate a comparison of the resulting kinetic and thermodynamic parameters obtained for the degradation of PVA. Although several kinetic methods are available, it is important to emphasize that the selection of model-free kinetic methods, as opposed to model-fitting methods, for estimating the kinetic data of this process is based on the recommendations of the International Conference on Thermal Analysis and Calorimetry (ICTAC) committee^31,32^. The committee compared several computational methods for estimating kinetic triplets (reaction model, activation energy and pre-exponential factor) and concluded that reliable estimations should be conducted using multiple heating rate programs (model-free methods), while the model-fitting single heating rate program should be deemed inappropriate. Furthermore, findings in^33,34^ suggest that a minimum of three heating rates is necessary to enhance the reliability of representing kinetic data.

**Fig. 4:**
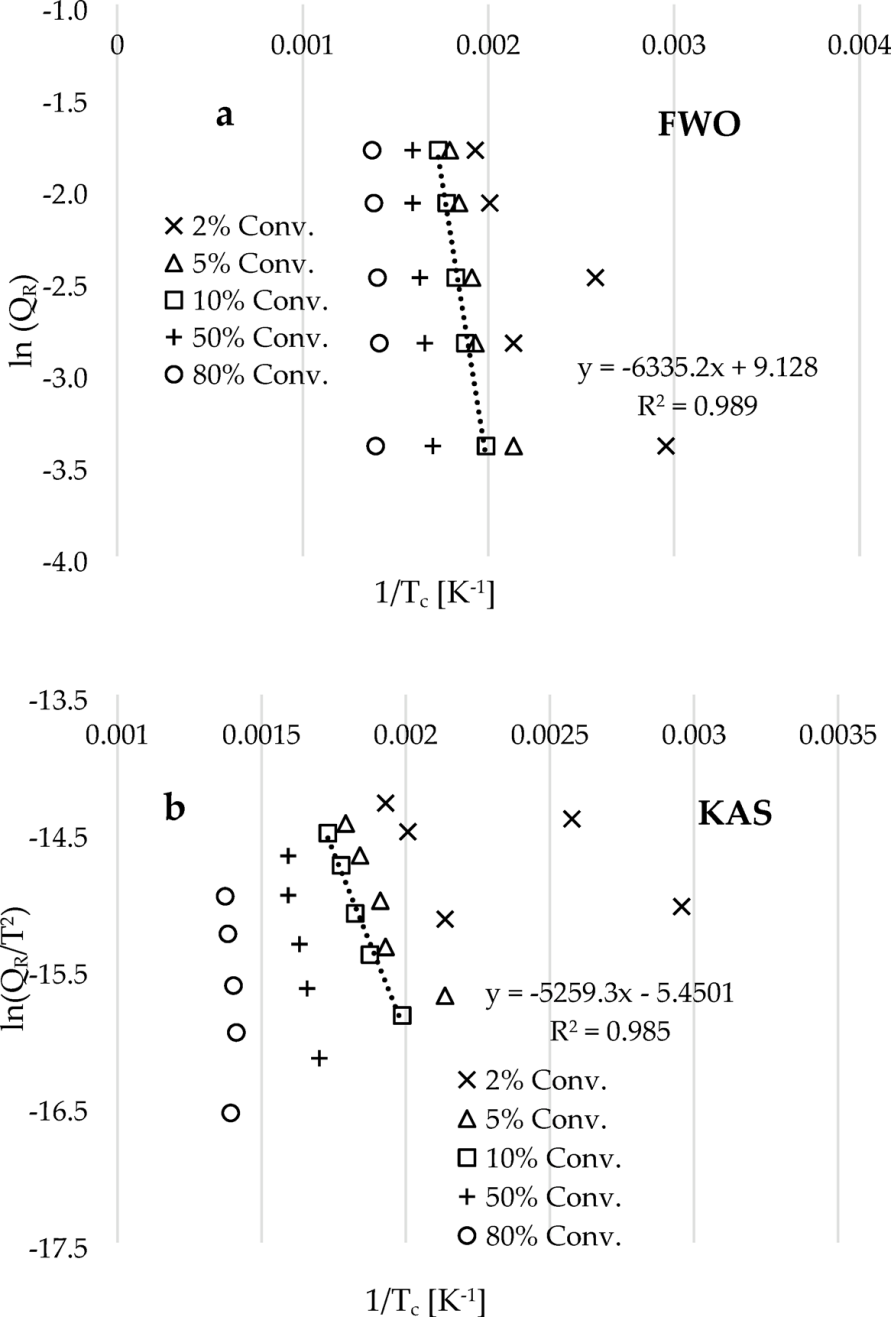
Kinetics plots of (**a**) $\:\text{l}\text{n}\left({Q}_{R}\right)$, and (**b**) $\:\text{ln}\left({Q}_{R}/{T}_{C}^{2}\right)$ against $\:1/T\:\left[{K}^{-1}\right]$ using the FWO and KAS kinetics model-free methods for variable heating rates, respectively.

Before estimating these kinetic and thermodynamic parameters, it is important to note that the TGA data curves of materials are typically categorized into three stages: dehydration, decomposition, and condensation^29,35,36^. Comparing with the literature^36–40^, it can be seen from Fig. 3c that there were minimal changes in weight observed in the five TGA traces at conversions below 5%. This indicates that this stage is classified as the dehydration phase. Holland and Hay^39^ reported that during the dehydration stage, PVA materials undergo degradation, resulting in the release of moisture and hydrolysis of volatile organic compounds such as methyl acetate and methanol. Following this stage, there is a significant decrease in material degradation due to the loss of material content and the transition from amorphous to crystalline phases with increasing temperature. This subsequent stage is referred to as the decomposition stage. Thomas et al.^40^ observed that at this elevated temperature, the scission of C-C bonds occurs during the degradation of PVA material. The study also revealed that volatile organic compounds are generated during this decomposition stage, leading to the formation of carbonyl end functional group products and small quantities of hydrocarbon-related products such as alkanes, alkenes, and aromatics. The final stage represents the complete crystalline phase, also known as residual ash or condensation phase, which can be observed in Fig. 3c occurring beyond 500 °C.min^−1^. Hence, the kinetic and thermodynamic parameters were determined estimated for constant conversion (2, 5, 10, 50, and 80%) and varied heating rate across the five TGA traces using the FWO and KAS model-free methods, as illustrated in Fig. 4a and b.

Figure 4a and b depict the graphs of $\:\text{l}\text{n}\left({Q}_{R}\right)$ and $\:\text{ln}\left({Q}_{R}/{T}_{C}^{2}\right)$ in relation to the inverse of conversion temperature ($\:1/T\:\left[{K}^{-1}\right]$) for the two chosen model-free kinetic models (FWO and KAS) under conditions of varying heating rate and constant conversion. Linear inverse relations were achieved for all the plots, and the corresponding slopes of these relations were utilized to estimate the activation energy and activation enthalpy, as presented in Tables 3 and 4. The tables indicate that the estimated values obtained from both kinetic models are comparable and exhibit an upward trend as the conversion increases. This ascending trend can be attributed to the temperature shift towards the maxima, resulting in an augmentation of degradation kinetics^41^ at higher conversions. The low values of $\:{E}_{A}$ estimated at low conversions, specifically at 2 and 5 wt%, indicate that the degradation reaction occurs more readily under these conditions and thus requires less energy to break the existing molecular bonds.

**Table 3:** Activation energy (E_A_) and activation enthalpy (ΔH) using FWO method for variable heating rates.

% Conversion	E_A_	*R*^2^ [-]	ΔH [kJ.mol^−1^]
	[kJ.mol^−1^]		
2	9.6±0.5	0.69	5.3
**5**	**36.6±1.8**	**0.92**	**32**
**10**	**50.1±2.5**	**0.99**	**45.3**
**50**	**107.2±5.4**	**0.99**	**102**
80	203.0±10.2	0.39	196.9
AVE	**81.3±4.1**		**76.3**

NB: The highlighted bold text indicates the conversion ranges that correspond to a higher coefficient of determination.

**Table 4:** Activation energy (E_A_) and activation enthalpy (ΔH) using FWO method for variable heating rates.

% Conversion	E_A_	*R*^2^ [-]	ΔH [kJ.mol^−1^]
	[kJ.mol^−1^]		
2	3.2 ± 0.2	0.18	−1.1
**5**	**30.1 ± 1.5**	**0.89**	**25.4**
**10**	**43.7± 2.2**	**0.96**	**38.9**
**50**	**102.6 ± 5.1**	**0.97**	**97.4**
80	201.6 ± 10.1	0.36	195.5
AVE	**76.2 ± 3.8**		**71.2**

NB: The highlighted bold text indicates the conversion ranges that correspond to a higher coefficient of determination.

The proximity of the estimated activation energies obtained through the FWO and KAS models is consistent with the findings substantiated in^26,27,42–45^ for various classes of polymeric and copolymeric materials. At a conversion rate of 2%, the activation enthalpy ($\:\varDelta\:H$) values obtained using both methods are notably low. The FWO method produces a value of 5.8 kJ·mol⁻¹, accompanied by a low coefficient of determination ($\:{R}^{2}=0.69$). A similar trend was observed at a higher conversion rate of 80%, which demonstrated a coefficient of determination of $\:{R}^{2}=0.39$. These weak correlations may be attributed to the overlaps observed in the TGA data curves at both low (initiation) and elevated (termination) temperatures. Huang et al.^46^ observed a similar significant increase in the thermal degradation of Cosmeceutical Benzoyl Peroxides containing Cu/Fe at low heating rates (1–10 °C·min⁻¹) at elevated temperatures, concluding that the ASTM 698 kinetic method reportedly yielded better correlations than the KAS method under these conditions. Nevertheless, the correlations obtained for the 5, 10, and 50 wt% conversion rates in this study were substantially stronger and approached unity, leading to estimated average activation energies of 64.6±3.2 kJ·mol⁻¹ and 58.9±2.9 kJ·mol⁻¹ for the FWO and KAS methods, respectively. Furthermore, the estimated average activation enthalpy values obtained for this range of conversions using the FWO and KAS methods were 59.7 kJ·mol⁻¹ and 53.9 kJ·mol⁻¹, respectively. This indicates that the application of the selected FWO and KAS model-free methods may be valid for conversions between 5 and 50 wt% loss in these pyrolysis operations.

Tables 3 and 4 illustrate that the estimated values of the activation enthalpy required for the formation of the activation complex are predominantly positive and exhibit an upward trend with increasing heating rates. The variation between the activation energy and enthalpy data ($\:R.{T}_{M}={E}_{A}-\varDelta\:H$) in these tables ranges from 4.3 to 6.1 kJ·mol⁻¹. These minor variations in $\:R.{T}_{M}$ indicate that the enthalpic barrier that must be surpassed for the reaction to proceed is closely aligned with the activation energy. The predominantly positive values of activation enthalpy suggest that the formation of activation complexes is an endothermic process, which is thermodynamically favourable^47^. In summary, the positive activation enthalpy values imply that the reactants possess a lower enthalpy than the transition state. Consequently, the decomposition reaction associated with thermal devolatilization (the breakdown of complex molecules into simpler ones^48^ of polyvinyl alcohol (PVA) under these operational conditions is non-spontaneous and necessitates an input of energy to disrupt the PVA polymer chains.

In comparison with relevant studies substantiated in the literature, Wulandari et al.^5^ estimated the activation energies ($\:{E}_{A}$) for PVA based on experimental TGA traces conducted at a heating rate of 10 °C.min^−1^. The activation energies were determined using the Coats-Redfern, Briodo, and Horowitz & Metzger kinetic models, yielding values of 57.1, 64.8, and 70.6 kJ.mol^−1^, respectively. For conversions ranging from 5 to 50 wt%, Table 2, and 3 present estimated values of 64.6 kJ.mol^−1^, and 58.81 kJ.mol^−1^ as the average activation energy obtained via the Flynn-Wall-Ozawa (FWO), and Kissinger-Akahira-Sunose (KAS) kinetic models, respectively. Any slight deviations in these values may be attributed to the differing conversion ranges employed in their study^5^, which were not explicitly stated in their work. In a previous study^2^, the average activation energy value for PVA material degradation was reported as 126 kJ.mol^−1^. This study^2^ utilized the CR model-fitting kinetic method and heating rates of 20, 30, and 40 °C.min^−1^, focusing on conversion rates between 10% and 70%. Additionally, a related publication by Peng et al.^24^ observed a corresponding shift in PVA traces to elevated temperatures with increasing heating rates from 10 to 50 °C.min^−1^, providing estimated activation energy values ranging from 120 to 170 kJ.mol^−1^ during the intermediate stage of pyrolysis, which extended to 280 kJ.mol^−1^ near the termination stage, or at higher conversions.

Sin et al.^41^ reported an average activation energy value of 128.6 kJ·mol⁻¹, while Peng and Kong^49^ estimated an activation energy value of 129.9 kJ·mol⁻¹ for the thermal degradation of polyvinyl alcohol (PVA) at degradation temperatures ranging from 100 to 600 °C, with heating rates between 10 and 50 °C·min⁻¹. Taghizadeh et al.^50^ observed that the presence of acetate groups in PVA samples results in a reduction in the rate of the primary reaction, thereby enhancing thermal stability as well as increasing the activation energy associated with the pyrolysis process. Taghizadeh et al.^50^ reported activation energy values of 191.0 kJ.mol^−1^ and 207.1 kJ.mol^−1^ for the pyrolysis of PVA(80) (hydrolysis grade > 80 mol%, and molecular weight of 9,000–10,000 g.mol^−1^) at the initiation (< 150 °C) and propagation stages (200–300 °C), respectively – utilising heating rates of 5, 15 and 25 °C.min^−1^. Fernandes et al.^23^ studied the pyrolysis of a polyvinyl alcohol (PVA) grade (98–99% hydrolysed, with a molecular weight of 13,000–23,000 g·mol⁻¹) under a nitrogen flow rate of 20 ml·min⁻¹. Additionally, they^23^ conducted a separate study under a synthetic airflow of 50 ml·min⁻¹, exploring heating rates between 2.5 and 20 °C·min⁻¹. The researchers^23^ utilized kinetic models derived from a reordering of the general Arrhenius equation, which yielded activation energies ranging from 75.0 to 137.0 kJ·mol⁻¹ in an oxidative environment and from 115 to 180 kJ·mol⁻¹ in an oxidation-free environment. It is noteworthy that both literature values were derived from TGA data obtained at elevated heating rates. However, it is essential to highlight that the average activation energy ($\:{E}_{A}$) values reported in these literature sources for polyvinyl alcohol (PVA) material are slightly higher than the average value estimated in this study. This observation implies that low heating rate pyrolysis may have led to a reduction in the thermal stability of the material, attributable to the extended residence time required to ensure the predominant influence of pyrolysis temperature on the PVA material.

Finally, it is essential to emphasize that the computed DNN data, along with the kinetic and thermodynamic data derived from the degradation of PVA at low heating rates, could provide critical insights for plastic manufacturers and environmental engineers. This information may enhance the accuracy of predictions regarding the thermal stability of this material, even in the absence of extensive experimental data, thereby facilitating the optimization of the thermal degradation process and the energy requirements associated with it. While the formulated model explicitly captures the behaviour characterized by the PVA data curves at low heating rates, the developed model is constrained by the range of data and process conditions considered in this study. Furthermore, the kinetic studies conducted in this research were grounded in the application of the FWO and KAS model-fitting kinetic models, bolstered by information obtained from the reports of the ICTAC committee^31,32,51^. The influence of pore-structure-related parameters, such as permeability, porosity, surface area, and mean particle diameter, along with other physical parameters, could be integrated into a DNN model trained to facilitate global predictions of TGA data curves. This integration may necessitate the training of extensive experimental datasets for several plastic materials prevalent in the environment. In addition, it is recommended to expand the scope of this study to include a broader range of heating rates for PVA traces, to explore various solid-state reaction mechanisms, to investigate alternative kinetic models, and to assess the potential applicability of agro-waste as a biocatalyst for the thermal degradation of different classes of polyvinyl alcohol (PVA).

## Conclusion

This study presents a comprehensive analysis of the TGA data curves obtained from the thermal decomposition of PVA material conducted at low heating rates (2, 5, and 10 °C.min^−1^) using machine learning deep neural networks (DNN). The DNN incorporates degradation time, degradation temperature, and heating input as input parameters. By employing this approach, a 2-hidden layer DNN framework was developed, with 8, 7, and 6 neurons in each layer. These frameworks facilitate the formulation of learning algorithms required for training the networks via backpropagation. The DNN framework with 8 hidden neurons was determined to offer the optimal computational time and accurate convergence at a reduced number of epochs. The computed values of synaptic weights and biases demonstrate the significant contribution of heating rate and degradation time to the overall output signals. Additionally, the learning algorithms successfully predicted TGA traces at 3.5 and 7.5 °C.min^−1^, which fell within the expected range of experimental values.

The activation energy and activation enthalpy for the TGA data were estimated using Flynn-Wall-Ozawa (FWO) and Kissinger-Akahira-Sunose (KAS) model-free kinetic methods for variable heating rates. The application of the FWO and KAS isoconvensional model-free methods resulted in average activation energies of 64.62 ± 3.23 kJ·mol⁻¹ and 58.81 ± 2.94 kJ·mol⁻¹, respectively. Additionally, the average activation enthalpies obtained through these two model-free kinetic methods were 59.73 kJ·mol⁻¹ and 58.81 kJ·mol⁻¹, respectively. The estimated values of activation enthalpy required for the formation of the activation complex for all selected kinetic models were predominantly positive, indicating an endothermic reaction.

This study provides a comprehensive application of deep neural networks and thermo-kinetic analysis for the thermal devolatilization of polyvinyl alcohol (PVA) plastic material, which is pertinent to plastic manufacturers, environmental engineers, and energy experts in quantifying the energy required for the end-of-life thermal degradation of PVA materials. It is noteworthy that further research could be conducted through the incorporation of biomass materials as feedstock to enhance the pyrolysis of PVA plastic. This proposed approach, intended for future investigation by this research group, could potentially identify the most suitable biomass materials and optimal operating conditions for co-pyrolysis operations, aimed at maximizing the production of bio-oil, bio-char, or thermally degrading to ash in a landfill.

## Data Availability

Data is provided within the manuscript. For more information, please contact Dr. AJ Otaru (email: aotaru@kfu.edu.sa).
